# Connecting the Dots Between Pharmacokinetics, Inflammatory Biomarkers, and Mucosal Healing in Patients on Infliximab with Inflammatory Bowel Disease

**DOI:** 10.3390/pharmaceutics18091146

**Published:** 2026-09-11

**Authors:** Ana Homšek Ilić, Marija Jovanović, Srđan Marković, Sandra Vezmar Kovačević, Tamara Knežević Ivanovski, Petar Svorcan, Radojka Savić, Katarina Vučićević

**Affiliations:** 1 Department of Pharmacokinetics and Clinical Pharmacy, Faculty of Pharmacy, University of Belgrade, 11221 Belgrade, Serbia; 2 Department of Gastroenterology and Hepatology, University Hospital Medical Centre “Zvezdara”, 11000 Belgrade, Serbia; 3 Faculty of Medicine, University of Belgrade, 11000 Belgrade, Serbia; 4 Department of Bioengineering and Therapeutic Sciences, University of California San Francisco (UCSF), San Francisco, CA 94143, USA

**Keywords:** therapeutic drug monitoring, mucosal healing, Crohn’s disease, ulcerative colitis, platelets, biomarkers

## Abstract

**Background/Objectives**: Mucosal healing (MH) is a key therapeutic target in inflammatory bowel disease (IBD) associated with sustained remission, reduced hospitalisation, and improved outcomes. Its assessment relies on endoscopic evaluation, which is invasive, costly, and not always feasible. Therapeutic drug monitoring (TDM) of infliximab (IFX) and evaluation of available biomarkers may provide non-invasive tools for predicting treatment response. This study investigated the association between IFX pharmacokinetics, routinely available blood biomarkers and MH to identify potential surrogates for predicting treatment outcomes. **Methods**: A monocentric, retrospective, real-world study was conducted, and pharmacokinetic analysis was performed in NONMEM version 7.5, applying a model with priors to estimate individual pharmacokinetic parameters. Endoscopic findings were collected, and patients were categorised according to achievement of MH. Univariate and multivariable logistic regression analyses were performed to evaluate the predictive value of tested variables, and receiver operating characteristic (ROC) curve analysis was performed to assess discriminative performance. **Results**: All evaluated variables demonstrated statistically significant associations with MH (*p* < 0.05). IFX clearance showed a strong inverse relationship with the probability of achieving MH. Leukocyte count exhibited particularly good discriminative ability, with an area under the ROC curve of 74.04% and an odds ratio of 0.78, highlighting its potential clinical utility. Multivariable logistic regression identified a model including IFX clearance and platelet count (PLT) as the most promising predictors of MH. Significant differences between patient subgroups indicated the association of higher PLT and modestly increased IFX clearance with a lower probability of MH. **Conclusions**: IFX clearance represents a promising surrogate marker for MH. TDM-derived parameters coupled with routinely available biomarkers may improve treatment response assessment. Combining pharmacokinetic and biomarker-based approaches could reduce reliance on repeated endoscopies while facilitating more effective IBD monitoring in clinical practice.

## 1. Introduction

Management of inflammatory bowel disease (IBD) remains a significant challenge in clinical practice. Infliximab (IFX), introduced for the treatment of Crohn’s disease (CD) in the late nineties [1], remains a highly effective option for a majority of patients. Still, around 10–40% of patients are non-responders to IFX, and even 30–60% of them experience loss of response (LOR) over time [2,3,4].

The goal of treatment in IBD remains the control of symptoms, such as rectal bleeding, stool frequency, abdominal pain, and stool consistency [5], as these can be easily assessed at each follow-up visit [6]. However, the primary endpoint in clinical studies and the goal in clinical practice for preventing disease progression is the achievement of mucosal healing (MH) in the affected parts of the gut [7]. The achievement of MH can be confirmed only through endoscopic evaluation, which allows direct visualisation of the intestinal mucosa and the application of disease-specific scoring systems. The use of standardised clinical scores has improved the objectivity of disease severity assessment and supports better control of inflammatory consequences; however, some of these scores remain insufficiently validated [8]. The most robust evidence for achieving and maintaining MH, compared to the other groups of drugs used in IBD, has been demonstrated for anti-TNFα monoclonal antibodies (mAbs) [7,8,9].

Endoscopic assessment of MH remains the gold standard for evaluating disease activity in patients with IBD. However, this method is invasive, uncomfortable for the patient, and is often resource-intensive, thereby limiting its feasibility and frequency in routine clinical practice [10]. Consequently, there is a need for more accessible, cost-effective, and reliable surrogate markers of disease activity [11]. Serological biomarkers are frequently used in clinical practice, as they are minimally invasive, routinely available, and can provide clinically relevant information related to disease activity and prognosis in patients with IBD [12]. However, they are nonspecific markers of systemic inflammation, and they support overall inflammatory status. Commonly used are inflammatory biomarkers, such as C-reactive protein (CRP), albumin (ALB) and erythrocyte sedimentation rate (ESR), as well as the faecal marker calprotectin (FCP) [10]. However, the correlation between endoscopic activity and inflammatory markers remains insufficient to support their widespread standalone use in patients with ulcerative colitis (UC) [13]. FCP and CRP are the most established biomarkers in CD patients; however, they have limitations for diagnosing MH or predicting treatment response, especially disease recurrence [12]. Although FCP has shown promising results [14,15,16], further research is needed to establish optimal monitoring strategies.

An increasing number of studies [10,17,18,19,20,21] have explored the use of platelets (PLT) in the assessment of IBD, either as count or volume or platelet-to-albumin ratio (PLT/ALB). Notably, one study demonstrated that patients with elevated baseline PLT count were less likely to achieve remission following IFX therapy, suggesting that PLT count may serve as a potential predictor of early treatment response [21]. Beyond their established role in haemostasis and thrombosis, PLTs are increasingly recognised as important mediators in inflammatory processes [18].

To date, the relationship between IFX pharmacokinetics (PK), inflammatory biomarkers, and the achievement of MH has not been fully elucidated. Here, we propose a real-world study to investigate how the interplay between IFX PK and stool- and/or blood-based biomarkers influences the likelihood of achieving MH.

## 2. Materials and Methods

### 2.1. Study Design and Participants

A monocentric, retrospective, real-world study was conducted between February 2023 and January 2025 at University Medical Centre “Zvezdara”, Belgrade, Republic of Serbia, one of the reference centres for IBD in the country. The research protocol received approval from the institutional Ethics Committee (University Medical Centre “Zvezdara”, No. IRB00009457, on 5 October 2022). The inclusion criteria for the study were: (1) patients older than 18 at check-ups (some patients were diagnosed before adulthood); (2) diagnosis of either UC or CD; (3) treatment with IFX; (4) at least one measured concentration at any time point during the treatment documented in the patient’s chart. Exclusion criteria were incomplete dosing and therapeutic drug monitoring (TDM) data in the medical records. Following model development, some patients were excluded from the treatment response analysis due to the absence of endoscopic assessment data in their medical chart.

### 2.2. Data Collection

For this research, data were collected from the hospital’s information system at the University Medical Centre “Zvezdara”. Data for each patient were documented in a release form, which was completed at every check-up, that is, every time patients received the next dose of IFX or in case of relapse. The form contains the following sections:

1. Patient characteristics (age, sex, body weight), time of hospitalisation and diagnosis;
2. Current disease and personal anamnesis with type of IBD, disease severity and phenotype, previous treatments;
3. Clinical state of the patient with the basic examination at admission (number of stools, consistency, blood, pain, other symptoms, information on LOR to treatment);
4. Commentary on the provided laboratory results with performed analysis of complete blood cell count, iron blood tests, biomarkers of inflammation and FCP.
5. Endoscopic disease assessment with appropriate scores depending on the type of IBD and radiography where applicable;
6. Therapy the patient received during hospitalisation;
7. Other drugs that the patient is using or is released with;
8. Planned control and the next dose administration.

Endoscopic assessment was carried out most commonly at week 52, with the interquartile range 52–82 weeks. Additionally, dosing regimen charts were reviewed. Each patient received the individualised dose per body weight of 5–10 mg/kg of IFX via two-hour infusion. The dose was calculated and rounded to the nearest hundred due to cost-effectiveness. In the induction phase, IFX was administered in weeks zero, two and six or, in an accelerated induction, every or every other week. After induction, patients received IFX every four, six or eight weeks as part of maintenance treatment. TDM was performed predominantly at weeks 6 and 14, with additional measurements at weeks 22, 46, or 52, as well as at clinically relevant time points during disease progression (at random time points). Median week of TDM was 46, with the interquartile range of 26–75 weeks. Due to the retrospective nature of the study, these time points have not been predetermined; however, the singled-out weeks had the most samples compared to the others. Additionally, the majority of samples were randomly gathered at patient check-up. Measurements were performed at the biochemical laboratory of the University Medical Centre “Zvezdara” using commercially available ELISA kits (R-Biopharm AG, Darmstadt, Germany) that were analysed on a Dynex DS2 analyser (Dynex Technologies, Chantilly, VA, USA). For some patients, when medical doctors decided it was necessary, anti-drug antibody (ADA) measurements were performed using the same approach as IFX with appropriate kits. From dosing regimen charts, the exact dose, date of administration, date of TDM sampling, and date of IFX treatment initiation were obtained.

### 2.3. Pharmacokinetic Model

After data collection, the final dataset contained sparse data with only trough concentrations of IFX. Since a great number of IFX PK models can be found in the literature, pharmacometric analysis was performed using the PRIOR approach, which allows the generation of stable PK models with sparse data with the help of knowledge from existing models. After identifying all available models at the time of research, each was assessed, and the ones containing enough information for this type of analysis were implemented in NONMEM^®^ version 7.5 (ICON Development Solutions, Ellicott City, MD, USA) [22] using the PRIOR subroutine as an alternative to fixing parameter values [23,24]. Model selection was based on a comprehensive evaluation of the predictive performance of models. Concentrations below the LLOQ (84; 5.34%) were handled with the M3 method [25]. Concentrations above the upper limit of quantification (81; 5.15%) fell outside the validated calibration range and could not be reliably quantified; these were re-assayed after dilution where feasible and retained, or otherwise excluded. Only IFX clearance and its variability were estimated. Normal-Inverse Wishart distribution subroutine was implemented, and the weight of the priors was calculated using Equation (1).

(1) $$df=2\cdot {\left(\frac{{OMEGA}^{2}}{{STANDARD\;ERROR}_{\left({OMEGA}^{2}\right)}}\right)}^{2}+1$$

Each existing error model (proportional, additive and combined) was applied to describe the residual variability; also, sigma priors were not implemented. After obtaining the base model, the effect of various covariates on clearance was assessed using a stepwise covariate model. A total of seven covariates were tested, which included: PLT, CRP, haemoglobin concentration, concomitant immunosuppressive drugs, clinical remission, treatment phase, and previous biologic treatment. This approach involves forward inclusion (*p* < 0.05) and backward elimination (*p* < 0.01) of covariates, guided by changes in the objective function value (OFV). In addition, covariate selection was guided by biological plausibility and the extent of reduction in inter-individual variability [26]. Pharmacometric nonlinear mixed-effects modelling analysis was performed in NONMEM^®^ together with a compiler using Perl-Speaks NONMEM (PsN, version 5.5.0). Pirana (version 24.9.2) was used to comprehend each model and compare them. R (version 4.5.1) and various packages (including tidyverse, ggplot2, dplyr, xpose) in RStudio (version 2025.09.1+401 “Cucumberleaf Sunflower”) were used for exploratory analysis and post-processing of outputs. Finally, for each patient, an individual PK profile was generated using the plot function, and the percentage of time during which IFX concentration remained above the therapeutic threshold of 7 μg/mL [27] was calculated by simulating individual concentration-time profiles for each patient based on individual PK parameter values and dosage regimens from medical charts. The simulation included three doses in the induction phase and seven doses in the maintenance phase.

### 2.4. Response to Treatment Analysis

In this part of the study, only patients with endoscopic assessment during IFX treatment were included. All computations and graph generation were performed in R (version 4.5.1) and various packages (including magrittr, ggplot2, dplyr, psych, tidyr, pROC, survival, patchwork, corrplot) in RStudio (version 2025.09.1+401 “Cucumberleaf Sunflower”). The primary treatment outcome was the achievement of mucosal healing (MH). Endoscopic disease activity was assessed using the Simple Endoscopic Score for Crohn’s Disease (SES-CD) in patients with CD and the Mayo Endoscopic Subscore (MES) in patients with UC. For pragmatic purposes, MH was defined as an SES-CD score < 3 in CD and an MES score of 0–1 in UC [28]. Additionally, for UC patients, histological remission was included (if available) to confirm MH.

Descriptive statistical analyses were conducted to characterise patient demographics, treatment, TDM data, and laboratory parameters. These analyses were performed for the overall MH dataset and stratified by treatment response to facilitate comparisons between patients who achieved MH and those who did not. Depending on the distribution of the variables, continuous data are presented as mean ± standard deviation for normally distributed variables or median with interquartile range for non-normally distributed variables. Normality was assessed using the Shapiro–Wilk test. Categorical variables are presented as frequencies with percentages. The number of missing data for each variable is given. Missing data were handled using complete-case analysis. Comparisons between patients with and without MH were performed using the Student’s *t*-test or the Mann–Whitney U test for continuous variables, and the χ^2^ test or Fisher’s exact test for categorical covariates, as appropriate. To identify significant predictors of MH, univariate and multivariable stepwise logistic regression were implemented. All variables that showed statistically significant differences between subgroups based on MH were tested (CL, trough concentration, ratio of clearance and trough concentration, median concentration until the event and week 104, percentage of time above threshold, previous biologic treatment, presence of anti-drug antibodies, LOR, laboratory remission, CRP, ALB, PLT, PLT/ALB, ESR, leucocyte count, FCP).

Odds ratios (ORs) with corresponding 95% confidence intervals (CI) were calculated to quantify the strength of association between predictor variables and MH. Receiver operating characteristic (ROC) curve analyses were performed to evaluate the discriminative performance of individual variables for predicting MH, and the areas under the curve (AUROC) were estimated. Discriminative ability was classified as acceptable, excellent, or outstanding, with AUROC < 0.7 considered to indicate no acceptable discrimination. Youden’s index was estimated to obtain the optimal cut-off value that maximises the difference between true positive and false positive rates. All *p*-values were two-sided, with statistical significance set at 5%.

## 3. Results

### 3.1. Pharmacokinetic Analysis

PK parameters were estimated for each patient in the overall dataset (N = 298 patients) using all available IFX concentrations in the database (Supplementary Table S1). The structural model was developed both without and with priors. A model without priors was unstable due to the sparsity of the concentration data. After thorough research, nine models had enough information to perform this type of analysis and calculate the weights of the priors [29,30,31,32,33,34,35,36,37]. The appropriate calculations were performed for each of the nine models, and priors were implemented in the codes. The most stable PK base model was obtained using priors from the Aubourg model [29], which demonstrated suitable overall performance (lower OFV, lower shrinkage and relative standard errors, diagnostic plots added as Figures S1–S3), giving a stable model; this model was also identified in the simulation-based normalised prediction distribution error diagnostics performed on the same dataset as the most suitable one [38]. After selecting the base two-compartment model, several covariates were tested. The backward selection process gave two statistically significant covariates: CRP and PLT. However, the effect of PLT on the typical value of clearance was less than 15% (7.7–13.7%), a commonly applied relevance threshold [39,40]. In addition, the impact of CRP was clinically meaningful only in a small subset of patients with extreme values (approximately 3% of the cohort). Moreover, a reduction in unexplained variability (0.5% on CL and 0.1% on central volume) implies that these factors are not a clinically relevant source of PK variability [26]. Finally, to avoid conflating PK effects with disease-state markers, and to facilitate interpretation of the observed PK–disease relationships, the structural PK model was developed based solely on concentration data. Although CRP and PLT were evaluated as potential covariates, neither demonstrated a clinically meaningful effect or a sufficient reduction in inter-individual variability to warrant their inclusion in the final PK model. Their close relationship with disease activity was also considered when interpreting their potential contribution to the PK model. Therefore, these covariates were not retained in the final model, and clearance estimates were derived from the base model. Final model parameter estimates are given in Table 1, and the appropriate visual predictive check is presented in the Supplementary Material (Figure S4). Additionally, NONMEM code is given in the Supplementary Material (File S1).

**Figure 1:**
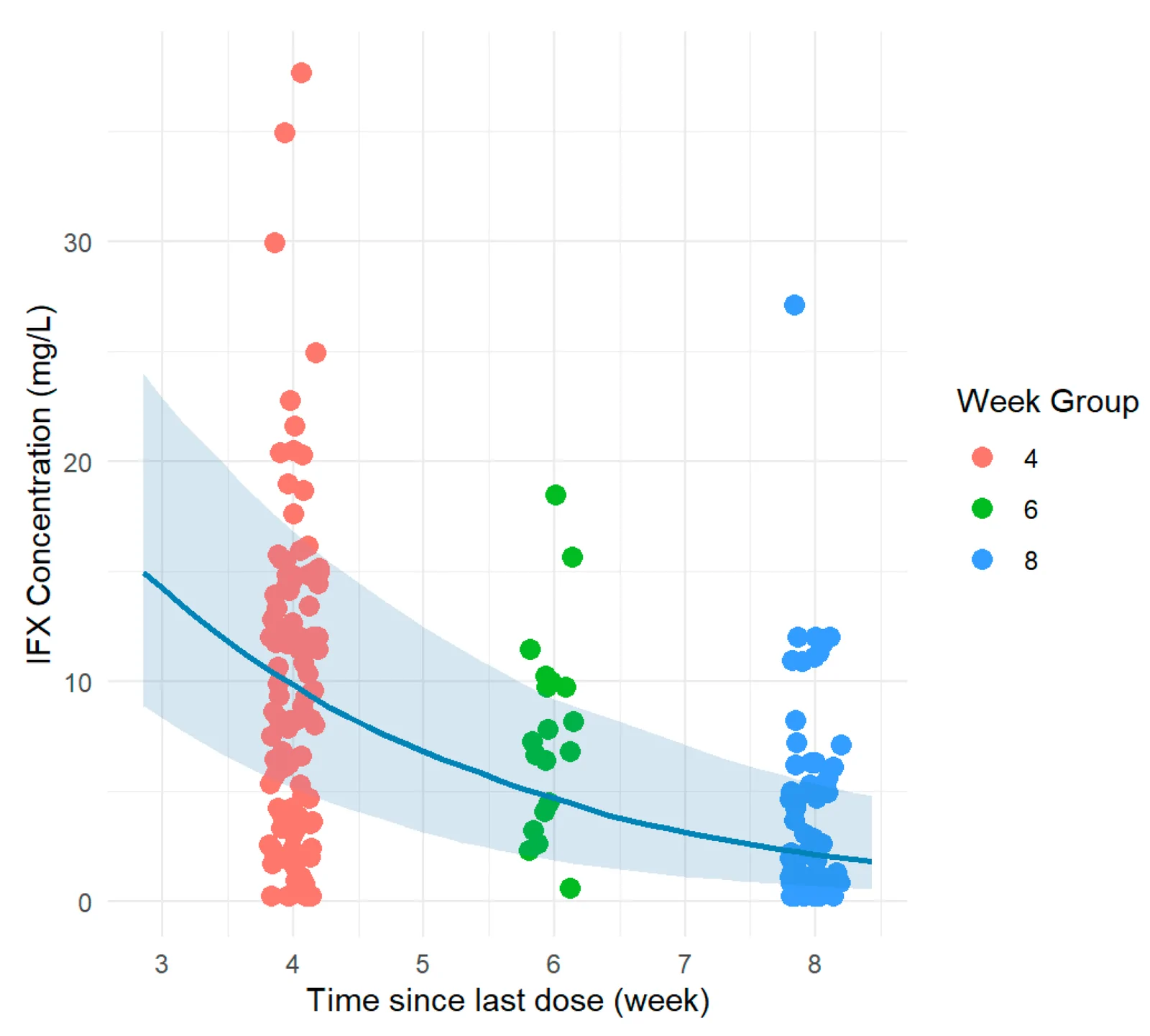
Observed infliximab (IFX) trough concentrations versus time after dose (weeks) overlaid with the population pharmacokinetic model-predicted median concentration–time profile. The shaded band represents the 5th–95th percentile prediction interval.

Using the obtained PK parameters for each individual, drug exposure profiles were generated. A target threshold of 7 μg/mL, based on treatment guidelines [27], was applied to evaluate the duration for which IFX concentrations remained above the target level. For each patient, the percentage of time above the assigned threshold was calculated from the reconstructed concentration–time profiles based on the obtained individual PK parameters and dosing regimen from the medical chart. Figure 1 depicts the entire cohort trough concentrations in the maintenance phase.

### 3.2. Outcome Analysis

For this part of the study, data from 248 IBD patients were selected from the overall datasets. Patients were excluded if no endoscopic assessment during IFX treatment was available. Demographic, treatment characteristics and biochemical parameters are presented in Table 2, Table 3 and Table 4. Depending on data distribution, variables are reported accordingly. The characteristics are further stratified into two subgroups based on the presence or absence of MH, and statistical significance was assessed by comparing each parameter between these subgroups.

IFX concentration measurements were gathered during treatment, at a time point closest to the assessment of the MH (CONC), with a median time distance of eight weeks (1–25). Additionally, all measured concentrations up until the event were accounted for, and the median was calculated (CONCMED), as well as the median for all concentrations during the 104 weeks (CALLMED), which has been chosen as the final endpoint (Table 2). As with the last concentration measured prior to the event (CONC), all biochemical parameters and clinical remission status were gathered at that time point (Table 3 and Table 4).

All IBD patients are pooled together, and no separate analysis of the two known types of diagnosis is presented, since no statistical significance between UC and CD cohorts was observed. Various PK parameters and exposure metrics, as well as clinical parameters, were subsequently evaluated and compared between patients who achieved endoscopic remission (MH) by week 104 and those who did not.

All tested variables demonstrated statistical significance (*p* < 0.05) in predicting MH (Table 5). IFX clearance had a strong negative effect on MH achievement, and the model performance fell into the acceptable range. Median IFX concentration until the outcome assessment had little to no effect on MH, with an even lower discriminative ability compared to drug clearance. The clearance-to-trough concentration ratio was evaluated as an exposure-adjusted marker of clearance. As IFX clearance increases with inflammatory and target-antigen burden while trough concentration correspondingly decreases, this ratio was hypothesised to reflect changes in disease-driven disposition more sensitively than clearance or concentration alone. It had a substantial effect, but model performance was poor. The effect size of PLT was negligible (OR ≈ 1.00), suggesting there was little practical impact on the treatment outcome. Although PLT count was indeed a statistically significant predictor, it demonstrated limited discriminative ability (AUCROC 66.56%). This suggests that while a relationship exists, PLT count alone is insufficient for outcome prediction, and additional predictors may be required to improve model performance. ALB level and PLT/ALB showed the highest discriminative performance with a significant effect, although with many missing values, this effect needs to be confirmed on a larger scale. Leukocyte count showed acceptable discriminative ability with an acceptable effect size, singling it out among tested variables, since the value was obtained for a great number of patients (66%). FCP and ESR both fell below the acceptable AUCROC, with no or rather small effect size. CRP had the lowest AUCROC, with a very small effect size, making it the weakest predictor among tested blood biomarkers. Conclusively, out of all tested PK and pharmacodynamic parameters influencing the probability of MH achievement, IFX clearance and PLT/ALB showed promising results, although their apparent predictive value requires confirmation in a larger cohort before it can be established.

**Figure 2:**
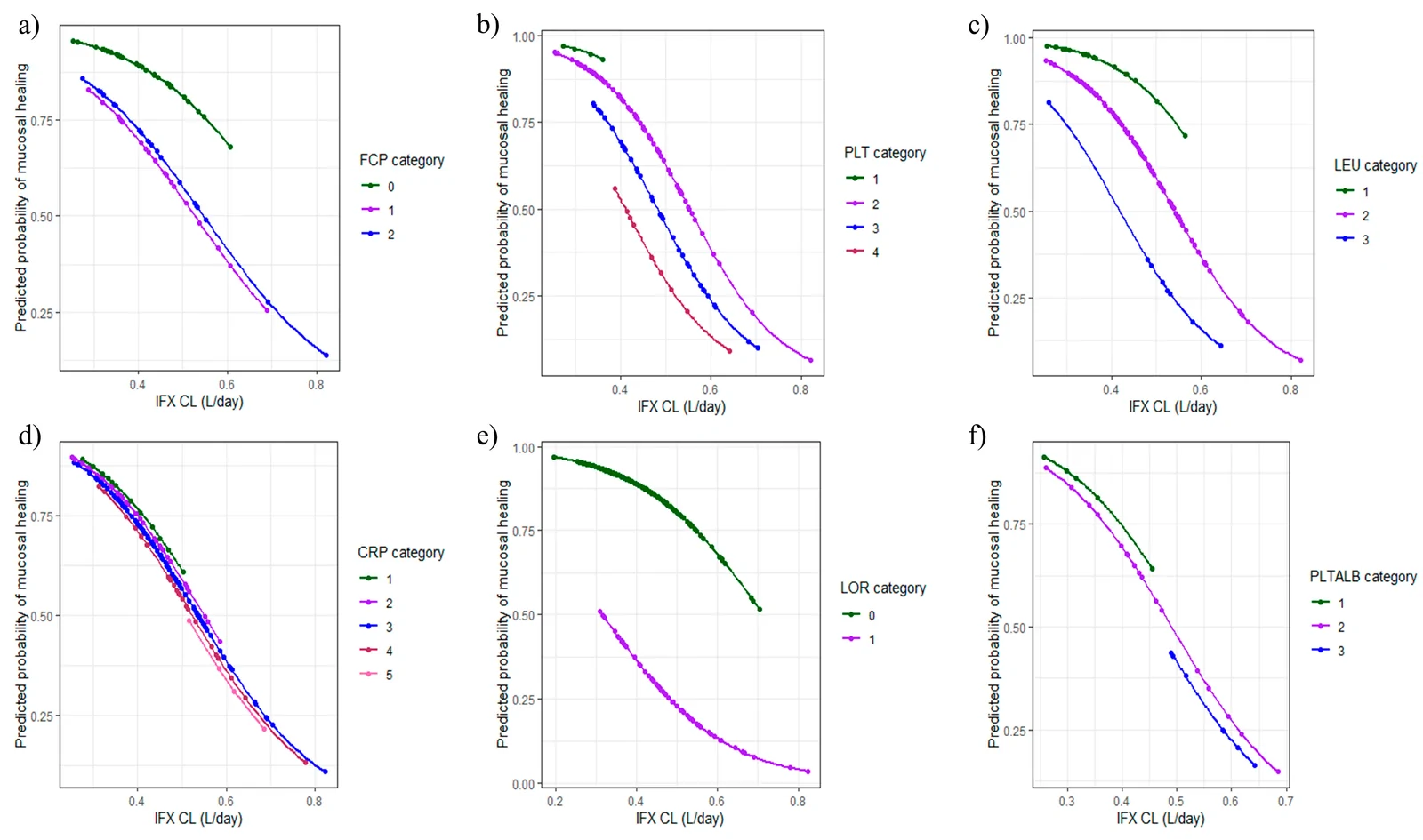
Multivariable logistic regression analysis of predicted mucosal healing (MH) probability based on infliximab (IFX) clearance and various predictors divided into categories: (**a**) faecal calprotectin (FCP), (**b**) platelet (PLT) count, (**c**) leukocyte (LEU) count, (**d**) C-reactive protein (CRP) level, (**e**) loss of response (LOR), (**f**) platelet-to-albumin ratio (PLT/ALB). Categories are explained in Table 6.

Finally, the continuation of the study included multivariable binary logistic regression of the probability of achieving MH, influenced by drug clearance as the most prominent PK parameter from univariate analysis and various biomarkers previously linked to response (Table 6 and Figure 2). Missing values were excluded from analyses. Biomarkers of response were divided into categories accordingly, based on the reference range for each one in the literature. For PLT level, an additional category was applied due to a threshold obtained in AUCROC analyses.

Each of the models included IFX clearance, as this parameter demonstrated superior performance in previous univariate logistic regression analyses. Given the inconclusive results for most dynamic clinical variables, several were further evaluated in combination with PK parameters to assess potential improvements in predictive performance (Table 6). Of the six models, three did not demonstrate satisfactory performance. In this patient cohort, FCP measurements and CRP levels were not identified as robust predictors of treatment response. No statistically significant differences were observed between patient subgroups stratified by these biomarkers. However, FCP measurements were unavailable in 70.56% of patients, limiting interpretability. CRP was missing in 21% of patients; nevertheless, the available data did not indicate a meaningful association with MH. Leukocyte count showed a potential predictive signal, with a *p*-value approaching 0.05 and clear separation between patient subgroups in the model. The most promising blood biomarker that was identified was PLT count. Although univariate analysis results were inconclusive, inclusion of PK-informed metrics improved overall model performance. Statistically significant differences were observed between subcategories, with higher PLT associated with a lower probability of MH and modestly increased IFX clearance.

## 4. Discussion

This study aimed to introduce the relationship between IFX PK, various blood-based inflammatory biomarkers, and MH in order to improve the efficiency of patient assessment in routine clinical practice. A substantial number of IFX PK models have been previously developed in patients with IBD. The estimated typical value of clearance (0.412 L/day) was comparable to several published models [29,32,41,42] and falls within the higher range of reported estimates. Our patient cohort consisted predominantly of patients on maintenance IFX treatment (85.1%), and a substantial proportion (35.9%) had severe disease, which may partly explain the higher observed clearance. As the dataset contained only trough concentrations, a PRIOR approach was used to obtain a stable model. Because troughs capture terminal elimination rather than the distribution phase, the data were informative for clearance (η-shrinkage 4%) but not for central volume (η-shrinkage 69%), whose individual estimates therefore contracted toward the population value. Inter-compartmental clearance and peripheral volume were characterised at the population level only. Interpretation is accordingly restricted to clearance and exposure, which drive the response analyses, and not to distribution parameters. Compared with the Aubourg et al. model [29], used for prior information, the estimated clearance lies between the reported values for males (0.456 L/day) and females (0.336 L/day). Covariates were explored to explain variability in IFX clearance (ranging from 0.1965 to 0.8221 L/day). Although CRP and PLT were statistically significant, their inclusion as covariates produced only a modest reduction in unexplained variability in clearance; this, together with the methodological considerations below, informed our decision not to retain them in the structural PK model. There is ongoing discussion regarding the preference for parameter estimation based on observed concentrations rather than relying primarily on covariate-driven models [43]. Multiple population PK studies of IFX have consistently demonstrated that a large proportion of inter-individual variability in clearance remains unexplained even after inclusion of routinely available clinical covariates, with reported reductions in unexplained variability often limited to modest improvements (typically below 20–30%). The primary aim of this study is to disentangle the independent and combined contributions of PK and inflammatory biomarkers to MH; incorporating biomarkers directly as covariates on IFX clearance could introduce structural confounding. This is particularly relevant because inflammatory biomarkers such as CRP and PLT count are not only potential determinants of disease activity, but are also dynamically influenced by IFX exposure, reflecting bidirectional relationships between inflammation and drug disposition [44]. To preserve interpretability and avoid conflating PK effects with disease-state indicators, the structural PK model was therefore kept parsimonious and based on concentration data. Biomarkers were subsequently evaluated as independent predictors of MH, allowing a clearer separation between drug exposure–driven effects and systemic inflammatory status, in line with contemporary model-informed precision dosing principles in IBD pharmacotherapy.

Several parameters depicting IFX PK have been tested as potential surrogate markers of response. In a review article [43], drug clearance was identified as the most promising PK-derived predictor of treatment response. More recent research [45] has further suggested that clearance in combination with trough concentration may provide improved predictive performance for clinical response. In this study, a clearance threshold of 0.26 L/day, together with a trough level of 5 µg/mL, has been proposed as indicative of treatment response. Our results suggest that higher IFX clearance (0.4876 L/day) is associated with the absence of MH. Nevertheless, our findings support IFX clearance as the most robust PK surrogate of MH among the evaluated exposure metrics. The second-best performing predictor was the clearance-to-trough concentration ratio assessed near the time of endoscopic assessment, with an optimal threshold of 0.1833. Other exposure metrics showed inferior performance, including trough concentration and median of concentrations prior to endoscopy, both showing OR values close to 1 and lower AUCROC values. The poorest performance was noticed, surprisingly, for the percentage of time above a predefined concentration threshold, a metric that theoretically reflects sustained drug exposure. Reported IFX trough concentrations associated with clinical response vary across studies. Early maintenance-phase (week 14) trough concentrations of approximately 2–3.5 µg/mL have been associated with treatment response or clinical remission [46,47,48,49]. Some authors suggest that targeting higher trough concentrations may improve the likelihood of achieving MH at endoscopic assessment [50,51,52,53]. In our study, the estimated threshold of 2.57 µg/mL measured close to endoscopy is in agreement with previously reported maintenance targets, a concentration of 2.3 µg/mL at the week of endoscopy [54]. Similarly, the slightly higher cut-off observed for the median of all troughs prior to endoscopy (4.45 µg/mL) aligns with findings by Imaeda et al. [51], who proposed a higher trough of 4 µg/mL to optimise MH outcomes.

Because endoscopic assessment is invasive, less acceptable to patients, and associated with increased healthcare costs, numerous efforts have been made to identify blood- or stool-based biomarkers that could serve as non-invasive surrogates of disease activity and MH [55]. FCP is the most widely used biomarker in IBD, with a large body of evidence supporting its association with intestinal inflammation [56,57,58,59,60,61,62] and its ability to reflect the extent of mucosal inflammatory burden [63]. Our results confirm that FCP demonstrated potential as a predictive biomarker, but it did not outperform other blood-based markers. Given that stool sampling is often less convenient than blood collection in routine clinical practice, there is growing interest in identifying reliable blood-based biomarkers of MH, as reflected in multiple recent studies [10,17,18,19,20,64]. Among the tested biochemical predictors, the most promising as a standalone marker was leukocyte count (OR 0.78, AUCROC 74.04%), consistent with findings from a previous machine-learning prediction model [65]. Good performance was observed for ALB level and PLT/ALB; however, these results should be interpreted with caution due to a substantial proportion of missing data (88.3%). Nevertheless, all tested blood biomarkers showed some degree of predictive ability for MH achievement. Although CRP has been proposed as a predictor of treatment response, particularly in UC rather than CD [66,67], it performed poorly in our study, likely reflecting its limited sensitivity and specificity in capturing IBD-related inflammatory activity. The literature on biochemical predictors of treatment outcomes and MH is heterogeneous, with varying and sometimes conflicting conclusions. Our study provides a clinically relevant perspective by evaluating biomarkers measured contemporaneously with IFX concentrations, thereby reflecting the drug exposure and inflammatory status of patients in close proximity to endoscopic assessment.

Consistent with previous findings, IFX clearance remained a significant predictor in all multivariable models, except PLT/ALB. We confirmed that patients with lower clearance were more likely to achieve MH and did not experience LOR, highlighting the clinical relevance of drug elimination as a key determinant of treatment success. Among routinely available biomarkers, PLT count in combination with IFX clearance emerged as the most informative predictor of response. PLT alone have been widely recognised as a valuable biomarker in IBD [18,68], either as count [21,69,70,71], volume [17,72], or ratio with another biomarker such as ALB [10,20]. In our analysis, CRP and PLT/ALB were not statistically significant, although there was a trend observed in PLT/ALB categories. Patients with a ratio < 5 tended to have lower clearance and a higher probability of achieving MH, whereas those with ratios > 10 showed the opposite pattern. However, due to the proportion of missing data, these findings should be interpreted cautiously, although they are consistent with previously published evidence [10,20]. For FCP, marginal statistical significance was obtained (*p*-value = 0.0866), with lower FCP levels (< 50 µg/g) generally associated with lower IFX clearance and a higher probability (around 70%) of achieving MH. However, this relationship was not consistent across all patients, limiting its predictive utility. Similarly, leukocyte count showed a trend toward significance (*p*-value = 0.0672), and a clear trend is visible in patients with leukocyte numbers outside the reference range. One patient in the high number subgroup had a lower clearance and achieved MH, and can be considered an outlier. These findings are broadly aligned with prior studies. For example, Qiu et al. identified, among others, PLT count, leukocyte count, ALB and IFX trough concentration as important predictors of long-term mAb efficacy using a machine-learning model [65]. Cao et al. demonstrated the relevance of trough IFX, CRP and FCP in predicting endoscopic remission [56], while Noh et al. highlighted the combined predictive value of FCP, ALB and CRP for deep intestinal healing [57]. Building on this evidence, our results provide additional insight into the interplay between IFX clearance and PLT count, supporting their combined use as a clinically meaningful approach for predicting MH in IBD.

A key strength of our study is the use of laboratory and TDM data obtained in close temporal proximity to endoscopic assessment. This approach enhances the clinical relevance of the findings, as it allows evaluation of whether routinely measured biomarkers can reliably reflect MH without the need for invasive procedures. Importantly, all evaluated predictors are routinely available in clinical practice. However, several limitations should be acknowledged. Firstly, the research is a single-centre study, which may limit the generalisability of the findings to broader IBD populations. Second, the retrospective design, based on routinely collected clinical data, resulted in missing values for several covariates. Third, TDM sampling was not standardised, and some trough concentrations were obtained at non-uniform time points without a predefined TDM protocol. Finally, although significant associations between IFX clearance, biomarkers, and MH were identified, the observational nature of the study precludes conclusions regarding causality. A key limitation is the absence of covariates in the model; their reliable estimation was precluded by the sparse, trough-only design and rather high volume of distribution η-shrinkage, and their omission may partly explain the slight over-prediction at the highest induction troughs. As multiple univariate comparisons were performed without correction for multiplicity, the reported associations should be regarded as exploratory and require confirmation in larger, independent cohorts. Further research is needed to validate the obtained results.

## 5. Conclusions

This study is, to our knowledge, the first to integrate individual, model-derived infliximab exposure metrics, obtained from sparse, trough-only real-world data using a PRIOR-based population PK approach with routinely available inflammatory biomarkers to predict endoscopically confirmed MH in IBD. Among all evaluated pharmacokinetic and inflammatory biomarkers, IFX clearance emerged as the most robust predictor of endoscopic healing. Routine inflammatory markers provided limited additional predictive value, although platelet count may warrant further evaluation alongside clearance estimates. These findings should be regarded as exploratory and require prospective validation in larger, independent cohorts before application to routine clinical decision-making. Nonetheless, they support the integration of TDM of IFX with routine assessment of blood-based biomarkers in predicting treatment outcomes, and with adequate longitudinal follow-up, such an approach may help reduce reliance on invasive endoscopic assessments.

## Figures and Tables

**Table 1:** Final infliximab (IFX) population pharmacokinetic (PopPK) parameter estimates using priors.

Parameter (Units)	Estimated Value (%RSE) / Shrinkage
CL (L/day)	0.422 (1.9)
Vc (L)	2.86 (3.4)
Q (L/day)	1.92 (3.3)
Vp (L)	4.58 (1)
IIV_CL_ (%)	23.5 * (1.7) / 4
IIV_Vc_ (%)	27.4 * (4.2) /69
Proportional residual error	0.593 (2.6)

* Values presented as approximate coefficient of variation. CL—clearance of IFX; IIV_CL_—inter-individual variability in clearance; IIV_Vc_—inter-individual variability in the central volume of distribution; Q—inter-compartmental clearance; V_c_—central volume of distribution; V_p_—peripheral volume of distribution; RSE—relative standard error.

**Table 2:** Demographic, pharmacokinetic (PK) and therapeutic drug monitoring (TDM) characteristics of infliximab (IFX).

	No MH (N = 77)	MH (N = 171)	Total (N = 248)
SEX			
Female	31 (40.3%)	72 (42.1%)	103 (41.5%)
Male	46 (59.7%)	99 (57.9%)	145 (58.5%)
WGT (kg)** **#	73.78 (13.44)	73.33 (12.54)	73.47 (12.79)
N-Miss	17	33	50
AGE (years)	36.0 (27.0, 45.0)	37.0 (26.5, 45.0)	36.5 (27.0, 45.0)
DOSE (mg)	400 (400, 500)	400 (300, 500)	400 (300, 500)
TAU (days) *	31 (28, 56)	49 (28, 56)	42 (28, 56)
PHASE			
Induction	9 (11.7%)	28 (16.4%)	37 (14.9%)
Maintenance	68 (88.3%)	143 (83.6%)	211 (85.1%)
CL (L/day) *	0.494 (0.412, 0.553)	0.412 (0.354, 0.474)	0.434 (0.364, 0.506)
Vc (L) *	2.77 (2.67, 2.92)	2.91 (2.79, 3.07)	2.87 (2.73, 3.04)
CONC (μg/mL) *	3.09 (0.84, 11.48)	7.21 (3.00, 12.00)	6.24 (2.21, 12.00)
N-Miss	4	20	24
CONCMED (μg/mL) *	3.97 (1.58, 9.09)	7.19 (3.43, 11.98)	6.83 (2.49, 11.44)
N-Miss	4	20	24
CALLMED (μg/mL) *	3.62 (1.40, 8.88)	6.86 (3.97, 10.95)	6.22 (2.90, 10.09)
PctTimeAbove (%) *	52.87 (41.90, 66.83)	61.10 (44.54, 76.06)	58.23 (43.32, 71.88)

# Variable was normally distributed and is presented as mean (standard deviation). All of the other continuous variables did not follow a normal distribution and are presented as median (first quartile, third quartile). * Statistically significant differences between subgroups (*p* < 0.05) were obtained with appropriate tests. CALLMED—median of all trough concentrations of IFX until week 104; CL—IFX clearance; CONC—trough concentration of IFX closest to mucosal healing assessment; CONCMED—median of trough concentrations of IFX up until the mucosal healing assessment; MH—mucosal healing; N-Miss—number of missing values; PctTimeAbove—per cent of time IFX was over the target concentration of 7 μg/mL; TAU—time since last dose; V_c_—IFX central volume of distribution; WGT—baseline weight.

**Table 3:** Results of the laboratory and biochemical analysis closest to the assessment of mucosal healing (MH).

	No MH (N = 77)	MH (N = 171)	Total (N = 248)
HGB (g/L) #	138.25 (15.97)	138.15 (16.46)	138.18 (16.25)
N-Miss	9	37	46
ER (10^12^/L) #	4.73 (0.47)	4.67 (0.56)	4.69 (0.53)
N-Miss	21	72	93
CRP (g/L) *	3.15 (1.10, 7.67)	1.46 (0.70, 3.78)	2.00 (0.89, 5.78)
N-Miss	7	45	52
ESR (mm/h) *	19.5 (10.25, 24.75)	9.0 (5.0, 16.0)	13.0 (6.0, 20.0)
N-Miss	43	128	171
LEU (10^9^/L) *	8.25 (6.19, 9.98)	6.40 (5.22, 8.17)	6.90 (5.5, 8.69)
N-Miss	33	76	109
PLT (10^9^/L) *	307.0 (251.0, 362.0)	260.0 (223.0, 298.0)	276.0 (227.0, 318.5)
N-Miss	32	88	120
ALB (g/L) *#	39.31 (4.89)	44.50 (4.68)	42.17 (5.37)
N-Miss	64	155	219
FCP (µg/g) *	222.40 (108.00, 694.00)	50.00 (26.50, 247.75)	119.00 (35.90, 339.00)
N-Miss	58	117	175
FCPCAT *			
<50	4 (21.1%)	29 (53.7%)	33 (45.2%)
50–250	7 (36.8%)	11 (20.4%)	18 (24.7%)
>250	8 (42.1%)	14 (25.9%)	22 (30.1%)
N-Miss	58	117	175

# Variable was normally distributed and is presented as mean (standard deviation). All other continuous variables did not follow a normal distribution and are presented as median (first quartile, third quartile). * Statistically significant differences between subgroups (*p* < 0.05) were obtained with appropriate tests. ALB—albumin level; CRP—C-reactive protein level; ER—erythrocyte count; FCP—faecal calprotectin level; FCPCAT—the categorical distribution of faecal calprotectin based on measured level; HGB—haemoglobin level; LEU—leukocyte count; MH—mucosal healing; N-Miss—number of missing values; PLT—platelet count; ESR—erythrocyte sedimentation rate.

**Table 4:** Treatment characteristics of patients.

	No MH (N = 77)	MH (N = 171)	Total (N = 248)
DX			
UC|CD	34 (44.2%)|43 (55.8%)	69 (40.4%)|102 (59.6%)	103 (41.5%)|145 (58.5%)
TIME (weeks) *	92.00 (42.00, 104.00)	52.00 (52.00, 52.00)	52.00 (52.00, 64.75)
ATI * (No|Yes)	69 (89.6%)|8 (10.4%)	165 (96.5%)|6 (3.5%)	234 (94.4%)|14 (5.6%)
IMM (No|Yes)	28 (36.4%)|49 (63.6%)	55 (32.2%)|116 (67.8%)	83 (33.5%)|165 (66.5%)
PREV * (No|Yes)	51 (66.2%)|26 (33.8%)	141 (82.5%)|30 (17.5%)	192 (77.4%)|56 (22.6%)
CLIN REMIS (NA| A)	20 (26.0%)|57 (74.0%)	37 (21.6%)|134 (78.4%)	57 (23.0%)|191 (77.0%)
SEV (No|Yes)	49 (63.6%)|28 (36.4%)	110 (64.3%)|61 (35.7%)	159 (64.1%)|89 (35.9%)
LOR * (1° and 2°|No)	48 (65.8%)|25 (34.2%)	17 (10.1%)|152 (89.9%)	65 (26.9%)|177 (73.1%)
N-Miss	4	2	6
LAB REMIS * (NA|A)	28 (38.9%)|44 (61.1%)	33 (23.1%)|110 (76.9%)	61 (28.4%)|154 (71.6%)
N-Miss	5	28	33

* Statistically significant difference between subgroups (*p* < 0.05) obtained with appropriate test. A—achieved; ATI—anti-infliximab antibodies; CD—Crohn’s disease; CLIN REMIS—clinical remission status; DX—diagnosis; IMM—concomitant treatment with immunomodulators; LAB REMIS—remission based on laboratory tests; LOR—loss of response; MH—mucosal healing; NA—not achieved; N-Miss—number of missing values; PREV—previous administration of biologic treatment; SEV—severity of IBD based on the presence fistulas or acute severe presentation; TIME—time of mucosal healing assessment; UC—ulcerative colitis.

**Table 5:** Univariate association of infliximab (IFX) pharmacokinetic (PK) parameters and inflammatory markers with mucosal healing (MH), combining group comparison, binary logistic regression, and ROC analyses.

Predictor Variable	N	*p*-Value	OR (95% CI)	AIC	AUROC (95% CI), %	Youden’s Index	Optimal Threshold
CL (L/day)	248	1.59 × 10^−7^	2.1 × 10^−4^ (7.2 × 10^−6^–4.5 × 10^−3^)	278.58	70.81 (63.75–77.87)	0.3219	0.4876
CONC (μg/mL)	224	0.001	1.04 (1.00–1.10)	283.39	63.42 (54.94–71.91)	0.2812	2.5735
CONCMED (μg/mL)	224	1.58 × 10^−3^	1.07 (1.01–1.13)	280.54	63.03 (54.9–71.16)	0.2438	4.4475
CL/CONC	224	6.51 × 10^−5^	0.31 (0.17–0.52)	264.7	66.47 (58.29–74.65)	0.3616	0.1833
PctTimeAbove (%)	248	0.0167	1.02 (1.00–1.03)	304.37	59.5 (52.09–66.91)	0.1912	68.4539
PLT (10^9^/L)	128	2.02 × 10^−3^	0.99 (0.99–1.00)	161.79	66.56 (56.48–76.64)	0.3248	298
PLT/ALB *	29	8.51 × 10^−3^	0.63 (0.40–0.89)	36.21	78.85 (62.06–95.63)	0.5337	6.9101
PLT * (10^9^/L)	29	0.0268	0.99 (0.98–1.00)	38.33	74.28 (56.04–92.51)	0.4712	294
CRP (g/L)	196	0.002	0.97 (0.94–0.99)	254.04	63.44 (55.42–71.46)	0.2381	2.055
ESR (mm/h)	77	0.006	0.94 (0.89–0.98)	102	68.43 (56.51–80.36)	0.3263	16.5
LEU (10^9^/L)	139	0.028	0.78 (0.56–1.02)	40.65	74.04% (54.65–93.43)	0.6106	7.3
ALB (g/L)	29	0.008	1.25 (1.06–1.57)	36.61	80.05 (63.9–96.19)	0.5481	44.5
FCP (µg/g)	73	0.010	1.00 (0.998–1.00)	85.33	69.83 (56.75–82.92)	0.3509	179.15

* Patients with albumin levels (11.7% of patients). *p*-value from Mann-Whitney U test; OR (95% CI) and AIC from univariate binary logistic regression; AUROC (95% CI), Youden’s index, and optimal threshold from ROC analysis. AIC—Akaike Information Criterion; ALB—albumin level; AUCROC—area under the receiver operating curve; CI—confidence interval; CL—IFX clearance; CL/CONC—ratio of IFX clearance and trough concentration measured closest to mucosal healing assessment; CONC—trough concentration closes to mucosal healing assessment; CONCMED—median of trough concentrations of IFX until the mucosal healing assessment; CRP—C-reactive protein level; ESR—erythrocyte sedimentation rate; FCP—faecal calprotectin level; N—number of observations; OR—odds ratio; PctTimeAbove—percentage of time IFX was over the target concentration of 7 μg/mL; PLT—platelet count; PLT/ALB—platelet-to-albumin ratio.

**Table 6:** Comparative presentation of models from multivariable logistic regression analysis.

Parameter (Unit)	Categories	Probability of MH	IFX Clearance	AIC	*p*-Value
FCP (μg/g)	0: <50	High	Low	79.518	Category 1: 0.0866 # Category 2: 0.1127
	1: 50–250	Any	Any		
	2: >250	Any	Any		
PLT (10^9^/L)	1: <150	High	Low	140.07	0.0288 *
	2: 150–300	Any	Any		
	3: 300–400	<80%	Any		
	4: >400	<60%	Higher		
LEU (10^9^/L)	1: <4.5	High	Low	147.5	0.0672 #
	2: 4.5–11	Any	Any		
	3: >11	Lower	Higher		
CRP (g/L)	1: <0.3	Any	Any	237.36	0.648
	2: 0.3–1				
	3: 1–10				
	4: 10–50				
	5: >50				
LOR	0: No LOR	>50%	Lower	211.85	8.19 × 10^−13^ ***
	1: 1° and 2° LOR	<50%	Higher		
PLT/ALB	1: <5	High	Low	38.448	0.7614
	2: 5–10	Any	Any		
	3: >10	Low	High		

In each model, drug clearance was a significant predictor (*p* < 0.05) except in the PLT/ALB model (0.071). AIC—Akaike Information Criterion; CRP—C-reactive protein; FCP—faecal calprotectin; LEU—leukocyte count; LOR—loss of response; MH—mucosal healing; PLT—platelet level; PLT/ALB—platelet-to-albumin ratio. Significance codes: #—marginally significant (0.05 *< p* ≤ 0.1); *—statistically significant (0.01 < *p* ≤ 0.05); ***—extremely significant (0 < *p* ≤ 0.001).

## Data Availability

The population pharmacokinetic models of infliximab used in this study were obtained from previously published peer-reviewed manuscripts. All relevant model details, including structural models, parameter estimates, and covariate effects, are available in the original publications cited in the reference list. The clinical data that support the findings of this study are not openly accessible due to privacy and confidentiality considerations related to the patients’ clinical data, and are available from the corresponding author upon reasonable request.

## Supplementary Materials

The following supporting information can be downloaded at https://www.mdpi.com/article/10.3390/pharmaceutics18091146/s1, Figure S1: Goodness-of-fit plots for the final model based on treatment phase; Figure S2: Distribution of ETAs for the final model. ETA1—variability on central volume of distribution, ETA4—variability on clearance; Figure S3: Distribution of random parameters for the final model. ETA1—variability in central volume of distribution, ETA4—variability in clearance; Figure S4: Visual predictive check of the developed two-compartment pharmacokinetic (PK) model of infliximab (IFX) with priors. Solid and dashed lines represent the median, 5th and 95th percentiles of the observed data with shaded 95% confidence intervals of the simulation-based prediction intervals; Table S1: Demographic characteristics of the dataset used to build a pharmacokinetic (PK) model of infliximab (IFX); File S1: NONMEM control stream for the final infliximab (IFX) population pharmacokinetic model.
