# Supplementary material for: Connecting the Dots Between Pharmacokinetics, Inflammatory Biomarkers, and Mucosal Healing in Patients on Infliximab with Inflammatory Bowel Disease
Source: Pharmaceutics. 2026 Sep 11;18(9):1146. doi: 10.3390/pharmaceutics18091146 (PMC13609954; doi:10.3390/pharmaceutics18091146)
Supplement: Supplementary file 1 [file pharmaceutics-18-01146-s001.zip › pharmaceutics-4503080-supplementary.pdf]

Article

Connecting the dots between pharmacokinetics, inflammatory biomarkers and mucosal healing in patients on infliximab with inflammatory bowel disease

Ana Homšek Ilić[1], Marija Jovanović[1], Srđan Marković[2,3], Sandra Vezmar Kovačević[1], Tamara Knežević Ivanovski[2,3], Petar Svorcan[2,3], Radojka Savić[4] and Katarina Vučićević[1*]

Table S1. Demographic characteristic of the dataset used to build a pharmacokinetic (PK) model of infliximab (IFX).

Characteristic [units/category]	Number (%)/Median (1st -3rd quartile)
Number of patients	298
Gender [male]	166 (55.7)
Age [year]	37 (27-45)
Body weight [kg]	75 (64-82)
Dose [mg]	400 (300-500)
Time since last dose [days]	39 (28-56)
Number of concentrations	1482
Trough concentration [mg/L]	6.49 (2.83-11.59)
Therapeutic drug monitoring samples	1236 (83.4)
Anti-drug antibodies [present]	57 (3.85)
Concomitant immunomodulators [yes]	865 (58.37)
Previous biologic treatment [yes]	73 (24.5)
C-reactive protein level [mg/L]	1.97 (0.8-4.7)
Albumin level [g/L]	42 (37-46)
Faecal calprotectin level [μg/g]	89 (50-385.5)

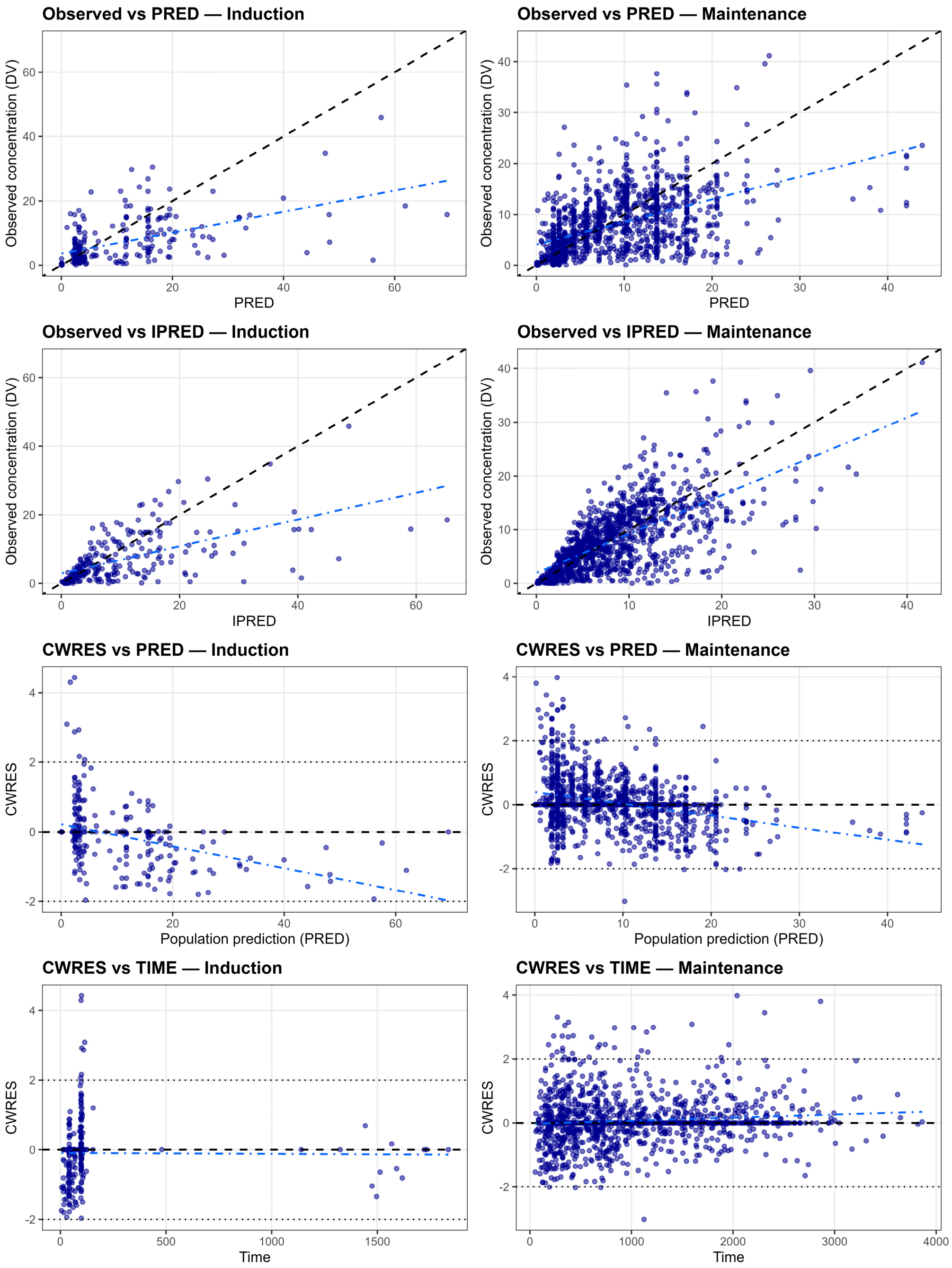

Figure S1. Goodness-of-fit plots for the final model based on treatment phase.

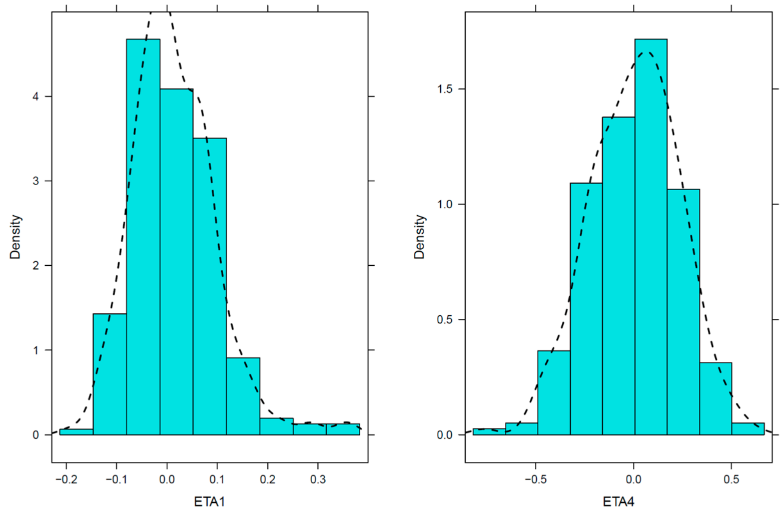

Figure S2. Distribution of ETAs for the final model. ETA1 – variability on central volume of distribution, ETA4 – variability on clearance.

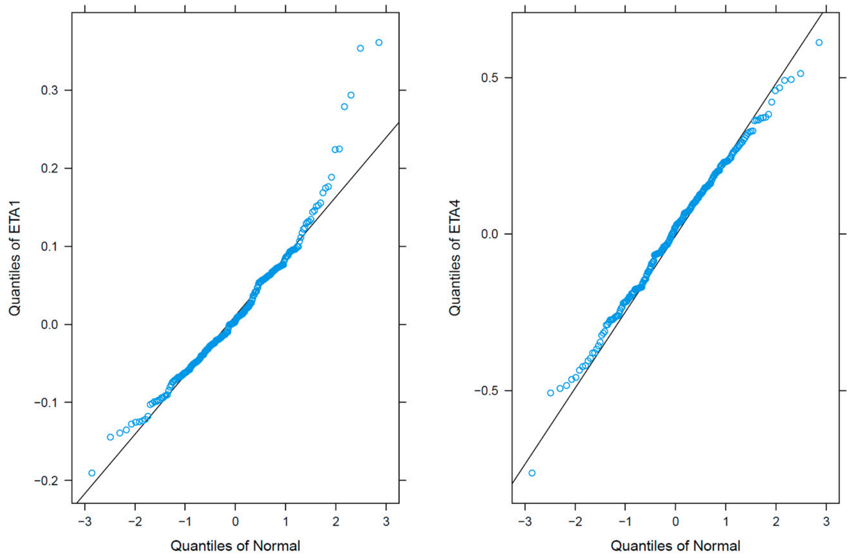

Figure S3. Distribution of random parameters for the final model. ETA1 – variability on central volume of distribution, ETA4 – variability on clearance.

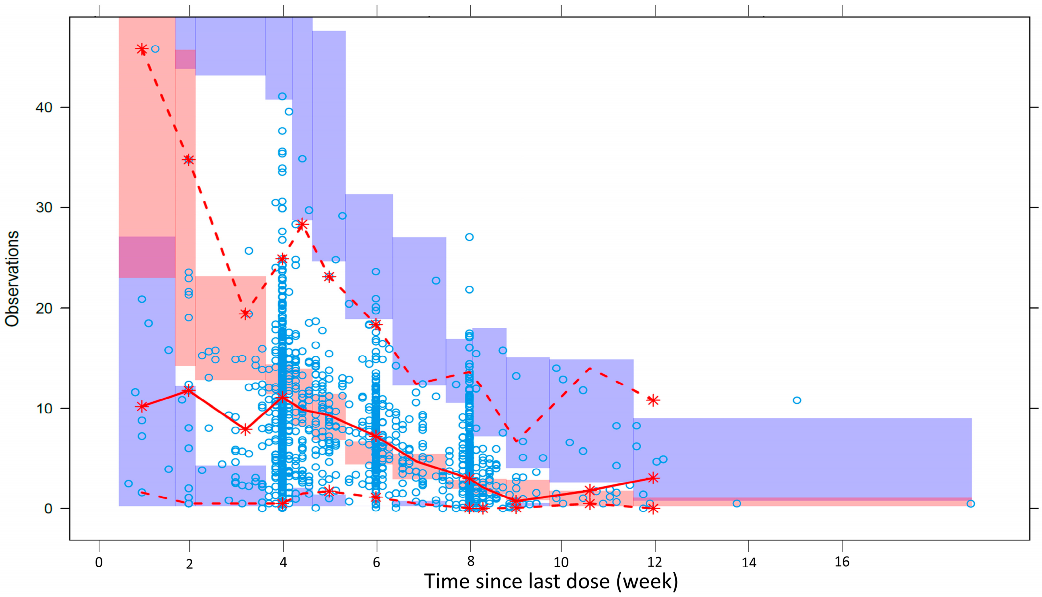

Figure S4. Visual predictive check of the developed two-compartment pharmacokinetic (PK) model of infliximab (IFX) with priors. Solid and dashed lines represent the median, 5th and 95th percentiles of the observed data with shaded 95% confidence intervals of the simulation-based prediction intervals.

File S1: NONMEM control stream for the final infliximab (IFX) population pharmacokinetic model

```
; ================================================================
; Final population PK model - infliximab (IFX) by Homsek, Ilic et al.
;
; Structure : 2-compartment, first-order elimination
;             (ADVAN3 TRANS4; CL, V1=Vc, Q, V2=Vp)
; Priors    : NWPRI (informative) on distribution parameters
;             V1, Q, V2 and on ETA(V1); CL estimated from the
```

```
;                   observed (trough) data - no prior on CL or ETA(CL)
; IIV          : estimated on CL and V1 (exponential);
;                   Q and V2 fixed to population values (no IIV)
; Residual   : proportional error (additive term fixed to 0)
; BLQ          : handled with the M3 method (Laplacian likelihood);
;                   LLOQ = 0.5 ug/mL
; ULOQ         : >ULOQ observations excluded prior to fitting
; Estimation: FOCE Laplacian INTERACTION (METHOD=1)
; ==============================================================
$PROBLEM      PK model IFX Homsek Ilic et al
$DATA         data.csv IGNORE=C
$SUBROUTINE ADVAN3 TRANS4         ; 2-compartment model
$PRIOR        NWPRI                    ; normal-Wishart informative priors

$PK
; ---- Clearance: estimated from data (no prior) ----
TVCL = THETA(4)
CL = TVCL * EXP(ETA(4))
; ---- Central volume ----
TVV1 = THETA(1)
V1 = TVV1 * EXP(ETA(1))
; ---- Inter-compartmental clearance (no IIV) ----
TVQ = THETA(2)
Q    = TVQ * EXP(ETA(2))
; ---- Peripheral volume (no IIV) ----
TVV2=THETA(3)
V2 = TVV2* EXP(ETA(3))
S1 = V1                                  ; scaling: central compartment
; ---- Micro-rate constants and disposition half-lives ----
K10 = CL/V1
K12 = Q/V1
K21 = Q/V2
SUM = K12 + K21 + K10
SSUM = SQRT(SUM*SUM-4*K10*K21)
ALPH = 0.5*(SUM+SSUM)               ; alpha (distribution) rate
BETA = 0.5*(SUM-SSUM)               ; beta (terminal) rate
HLAL = 0.693/ALPH                   ; distribution half-life
HLBE = 0.693/BETA                   ; terminal half-life
AUC=AMT/CL                          ; dose-interval AUC

$ERROR
IPRED = F
W = SQRT(THETA(5)**2*IPRED**2 + THETA(6)**2)     ; prop + add SD (add fixed to 0)
LLOQ=0.5                             ; lower limit of quantification (ug/mL)
IRES = DV-IPRED
IWRES = IRES/W

; ---- Quantifiable observations ----
IF (BLQ.EQ.0) THEN
F_FLAG=0
Y = IPRED + W*EPS(1)
```

```
ENDIF

; ---- BLQ observations: M3 method (likelihood-based) ----
IF (BLQ.EQ.1) THEN
F_FLAG=1
DUM=(LLOQ-IPRED)/W
CUMD=PHI(DUM)                    ; P(obs < LLOQ)
Y=CUMD
ENDIF

$THETA
 (0,2.81) ; V1   - central volume (L)
 (0,1.94) ; Q    - inter-compartmental clearance (L/day)
 (0,4.56) ; V2   - peripheral volume (L)
 (0,0.426) ; CL - clearance (L/day), estimated from data
 0.571 ; Proportional residual error (SD)
 0 FIX ; Additive residual error (SD) - fixed to 0

$OMEGA
 0.0713 ; IIV V1
 0 FIX   ; IIV Q   - no IIV (not identifiable from trough data)
 0 FIX   ; IIV V2 - no IIV (not identifiable from trough data)
 0.0483 ; IIV CL

; ---- Prior specification (NWPRI): distribution parameters only ----
$THETAP
 2.6 FIX     ; prior mean V1
 1.992 FIX ; prior mean Q
 4.5 FIX     ; prior mean V2

$THETAPV
 0.01082 FIX   ; prior variance V1
 0.00357 FIX   ; prior variance Q
 0.002025 FIX ; prior variance V2

$OMEGAP 0.0729 FIX ; prior ETA for V1

$OMEGAPD 313.5 FIX ; prior df for V1 OMEGA

$SIGMA   1 FIX

$ESTIMATION METHOD=1 LAPLACIAN INTERACTION NUMERICAL SLOW
            MAXEVALS=9990 NOABORT SIG=3 PRINT=5 POSTHOC
            MSFO=run1000.msfo
```
